# Glasgow Royal Infirmary

**Published:** 1832-12

**Authors:** 


### GLASGOW ROYAL INFIRMARY.

*Cases of Aneurism.* (From Dr. MACFARLANE'S *Clinical Reports.*)

### *Popliteal Aneurism; Ligature of the Femoral; Hemorrhage on the nineteenth Day.*

J. F., æt. forty-two, farm-servant, was admitted on the 5th July, 1826. Five weeks before, he received a kick in the left ham, which produced severe pain, impeded his walking, and prevented him from following his usual employment. After two weeks, he became sensible of a tumor in the ham: when the pain of the leg increased, and was combined with a feeling of numbness about the foot and ankle. The tumor, which pulsated distinctly, and could be emptied by pressure, was ill defined, and situated between the origins of the gastrocnemius externus. The leg was swollen, œdematous, and deformed about the centre of the tibia, where he had sustained a compound fracture fifteen years ago. His general health was good, and the pulse seventy-six.

On the 9th, the femoral artery was exposed at the inner edge of the sartorius, and tied with a single ligature. The external incision required to be larger than usual, on account of the great quantity of adipose substance, both under the integuments, and between the sartorius muscle and the sheath of the femoral vessels.

During the first twenty-four hours, the temperature of the left leg and foot was nearly two degrees higher than the opposite one. The limb was free from pain, swelling, and tension; and the superficial veins were prominent and well filled. Little or no febrile excitement took place: the wound adhered, except where the ligature passed out: the tumor became firm and incompressible, but diminished slowly.

On the 28th, at seven A.M., when attempting to get out of bed, a sudden gush of arterial blood took place from the wound, by the side of the ligature. It flowed, per saltum, to the amount of about eight ounces, and was arrested by pressure. A tourniquet was applied a little above the wound, and retained for a few days; whilst rest in the recumbent position was strictly enjoined. The ligature did not separate till the 4th of August, being the twenty-sixth day from the operation. There was no return of hemorrhage: the wound gradually closed, and he was dismissed cured in the beginning of September.

The aneurism was in this case to be attributed to external injury, the blow having probably lacerated the internal and middle coats of the artery; by which means the external yielded to the impetus of the circulation, and a sac was formed. The hemorrhage, which evidently proceeded from the femoral artery, shewed that this vessel was not completely closed; and there was, therefore, a risk of its recurring as long as the ligature remained attached. It was much longer of separating than usual; but, as the bleeding did not return, no ulterior measures were required.

*Aneurism of the right Popliteal and anterior Tibial Arteries; Ligature of the Femoral. Return of Pulsation in both Tumors on the twelfth Day.*

A. P., aged thirty-eight, farmer, applied to me for advice in the waiting room of the Infirmary, on the 12th November, 1826, on account of two tumors in the right leg. He did not intend to become a patient in the hospital, but, on learning that an operation would be required, he promised to remain in town, and requested my attendance. He was a healthy and robust man, and first observed a small pulsating tumor on the fore part of the right leg, about six inches below the knee. It was exactly over the course of the anterior tibial artery; seemed deep seated, ill defined, and about the size of a walnut. It pulsated distinctly, and could be partially diminished by pressure; and its pulsations were readily arrested on compressing the femoral. About five months after the appearance of this tumor, which he attributed to a blow he had received on the part, he began to complain of pain and stiffness in the knee, which gradually produced lameness. He then discovered a tumor in the ham, about tbe size of a pigeon's egg; which, at the time I saw him, was as large as an orange, and had all the characters of an aneurism. The foot and leg were slightly œdematous; and the pain, which had latterly extended to these parts, was so excruciating, that he was often obliged to assume a recumbent position, and to elevate the leg considerably above the level of the body, before it was mitigated. The pulse was natural; and, after a careful examination of the heart and large arteries, no other disease could be discovered.

On the 15th, I proceeded to expose the right femoral artery in the upper third of the thigh, where it is crossed by the sartorius, and passed a single silk ligature around it. The moment this was firmly secured, the pulsations of the tumors ceased: both ends of the ligature were cut off close to the knot: the edges of the wound were brought together by adhesive plaster, and a few turns of a bandage applied to the thigh.

Every thing went on favorably: the heat of the limb was preserved, the œdema speedily disappeared, the pain was only slight and occasional, the tumors became softer and smaller, the wound adhered by the first intention, and the febrile excitement was moderate.

On the 27th, I was hurriedly sent for in the morning, the patient having been alarmed during the night on discovering that both tumors were pulsating. He had been induced to lay his hand on the tumor in the ham, from having felt a return of the painful sensations about the knee, similar to those he experienced previous to the operation; and he stated that the pulsation of the popliteal tumor was then distinct, but that two hours elapsed before he could discover it in the anterior tibial one. Both tumors had regained their former size, but the pulsation was more obscure in the lower than in the upper one; in both, however, it could be easily stopt by compressing the inguinal artery. As his pulse was full and strong, I immediately bled him to syncope, ordered the frequent use of nauseating doses of emetic tartar, applied a graduated compress and a roller to each tumor, over which two small bags, composed of waterproof cloth, and half filled with pounded ice, were secured. These were repeatedly renewed, and a great degree of cold produced, without the slightest pain or inconvenience to the patient.

On the 4th day, I removed the compresses, in order to ascertain if the pulsation still continued. I was gratified to find that it had completely ceased in both tumors, which were somewhat flattened by the pressure, and had a firm feel. The compresses and bandage were re-applied, though less firmly: the bags of ice were discontinued, but the antiphlogistic treatment was persisted in for several days longer. He was kept in bed, and on spare diet, till the middle of January; by which time the tumors had diminished more than one half, and he was greatly reduced in flesh and strength. When I saw him last, about a year ago, he was in excellent health, and the remains of the aneurismal tumors could hardly be discovered.

The fact that more than one aneurism may exist in the same individual, unconnected with general disease of the arterial system, is obvious from the detail of the preceding case. The causes which seemed to give rise to the formation of both the anterior tibial and popliteal tumors, were of a local kind; and the case was more favorable for operation than the one which I first detailed. There was some reason to suppose, however, from the existence of two aneurisms, so near to each other in the same limb, that there was a partial disposition to disease in the coats of the affected vessels. But had the arteries of the limb been generally diseased, an occurrence which the age and robust health of the patient rendered improbable, the ligature of the femoral would have been followed by imperfect collateral circulation and gangrene.

When aneurism of the anterior tibial artery is situated in the upper part of the leg, the circulation of blood through it is generally commanded by compression of the femoral, and ligature of the latter vessel is usually successful in effecting a cure. The return of the circulation to both tumors on the twelfth day after the operation, does not militate against this opinion. The pulsation first commenced in the popliteal tumor, where it continued for two hours before it was discovered in the anterior tibial one; it must, therefore, have been owing not to a direct communication with any large collateral branch proceeding from the upper part of the thigh to the leg, but to the existence of some large arterial branch, originating higher up than the place to which the ligature was applied, and terminating in or close to the popliteal sac. The same thing happened to the late Dr. George Monteath, of this city, in a case of popliteal aneurism; but in his patient the pulsation was much longer of returning after the femoral was tied, and the cure was more protracted. Sir E. Home, in detailing some of the cases operated upon by Mr. Hunter, also states that the popliteal tumor frequently continued to pulsate after the femoral was tied; but that this circumstance did not generally interfere with the cure of the disease; as he found that, in most instances, a cure was produced by simply diminishing the force of the circulation through the aneurismal artery, without its being altogether obstructed. I was unwilling, however, to trust to the occurrence of this natural termination in the treatment of the case which I have now detailed: and I therefore proceeded, as soon as the return of the pulsation was discovered, to subdue it by local and constitutional means, which were fortunately successful.

Of late, an attempt has been made by Mr. Wardrop to revive the operation of Brasdor for the cure of aneurism. This operation, which consists in the application of a ligature on the distal, instead of the cardiac, side of the sac, was recommended and practised several years ago, both in this country and in France; but the result was uniformly fatal. It has since succeeded in the hands of of Mr. Wardrop; but it appears very doubtful if it will ever become one of those established operations to which we can confidently have recourse in cases where the Hunterian method is inadmissible. I have no intention of entering here into a detailed examination of its merits or demerits: I have only been induced to allude to the subject, for the purpose of shortly stating a case of aneurism at the root of the neck, for the cure of which I was strongly urged, by several of my medical friends, to have recourse to Brasdor's operation. I declined to follow this advice, because I could not satisfy myself, even after repeated and careful examinations, of the particular vessel implicated in the disease.

